# Fast genomic μChIP-chip from 1,000 cells

**DOI:** 10.1186/gb-2009-10-2-r13

**Published:** 2009-02-10

**Authors:** John Arne Dahl, Andrew H Reiner, Philippe Collas

**Affiliations:** 1 Institute of Basic Medical Sciences, Department of Biochemistry, Faculty of Medicine, University of Oslo, 0317 Oslo, Norway

## Abstract

Genome-wide location analysis of histone modifications and transcription factor binding relies on chromatin immunoprecipitation (ChIP) assays. These assays are, however, time-consuming and require large numbers of cells, hindering their application to the analysis of many interesting cell types. We report here a fast microChIP (μChIP) assay for 1,000 cells in combination with microarrays to produce genome-scale surveys of histone modifications. μChIP-chip reliably reproduces data obtained by large-scale assays: H3K9ac and H3K9m3 enrichment profiles are conserved and nucleosome-free regions are revealed.

## Abstract 2

A new method for rapid genome-wide μChIP-chip from as few as 1,000 cells.

## Background

Chromatin immunoprecipitation (ChIP) has been widely used to analyze the location of post-translationally modified histones or transcription factors in a genome *in vivo *[1-4]. ChIP analysis of DNA-protein interactions has led to significant advances in the understanding of gene regulation and of how epigenetic phenomena are regulated to affect gene expression, DNA repair and replication [5,6]. In a typical ChIP assay, large numbers of cells are used, DNA and proteins are cross-linked and chromatin is sheared to fragments of approximately 400-500 bp. Antibodies to the protein of interest are coupled to beads and used to pull down protein-DNA complexes. Chromatin is eluted from the complexes, cross-links are reversed and ChIP DNA is purified. A limited number of genomic sequences associated with the precipitated protein can be identified by PCR. Alternatively, high-throughput sequencing or hybridization to DNA microarrays (ChIP-chip) enables genome-scale mapping [7].

The range of biological applications of ChIP assays has been limited by the requirement for large cell numbers (approximately 10^7 ^cells per immunoprecipitation) and the length of the procedure (typically 3-5 days). To remedy to these limitations, a few ChIP-PCR strategies have recently been reported. A 'carrier ChIP' protocol [8] entails immunoprecipitation of chromatin from 100-1,000 mouse cells by mixing with millions of *Drosophila *cells; however, the assay takes several days and is unsuitable for genome-wide analysis due to excess of *Drosophila *carrier DNA that would interfere with such analysis. A one-day 'fast ChIP' assay [9] simplifies the procedure but has only been demonstrated for large cell samples and PCR assessment of relatively few loci. We have reported a downscaled Q^2^ChIP assay [10] for analysis of multiple proteins in 100,000 cells and, subsequently, a microChIP (μChIP) protocol [11,12] for as few as 100 cells. Again, however, only few loci could be examined with these procedures. Concomitantly, another microChIP assay was reported for 10,000-100,000 cells, which allows genome-wide analysis by ChIP-chip [13]. This assay represents an advancement in ChIP applications, but it remains labor intensive, takes over 4 days and has been validated for 100,000 cells and the top 30% of enriched promoters only. Except for this single attempt to downscale the genome-wide approach, ChIP-chip typically starts out with 10^7^-10^8 ^cells and yields amplified DNA ready for labeling and hybridization after 4-5 days. Our ultimate goal is to enable genome-scale investigation of histone modifications in very small cell samples, such as sorted stem cell populations, human tumor biopsies and embryonic cells. Therefore, we wished to move beyond these limitations, reduce the cell numbers and time required, and enhance the robustness of the assay.

We report here the optimization and validation of a one-day μChIP assay that enables genome-wide surveys of epigenetic histone modifications from 1,000 cells using microarrays. Typically, reliable resolution of ChIP location analysis is ensured through gel electrophoresis assessment of a sample of the fragmented chromatin to determine average DNA fragment length. However, this would require many more cells than what we used in this study. Thus, we devised a PCR-based approach and formulated an equation to allow an estimation of chromatin fragmentation in small cell samples, a step critical for reliable resolution of μChIP-chip analysis. Using μChIP-chip, we investigated the enrichment, on promoter regions, of acetylated lysine 9 and trimethylated lysine 9 of histone H3 (H3K9ac and H3K9m3, respectively) associated with transcriptionally active and silent promoters [14]. Four distinct classes of genes were identified based on differential marking by these modifications. μChIP-chip also demonstrates a nucleosome-free region immediately upstream of the transcription start site (TSS) for active genes, and shows that silenced genes exhibit a more closed chromatin conformation. Furthermore, construction of a metagene and correlation analysis reveal mutually exclusive occupancy profiles for H3K9ac and H3K9m3.

## Results and discussion

### Optimization and validation of μChIP-chip

**Figure 1:**
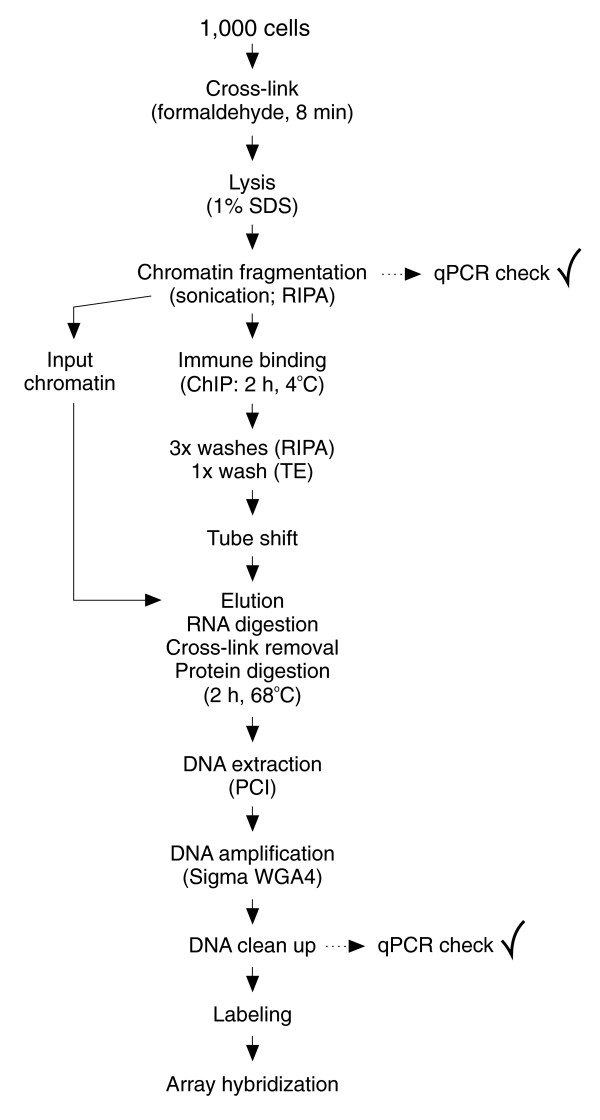
Flow chart of the μChIP-chip procedure.

We established and validated μChIP-chip (Figure 1) by monitoring promoter association of H3K9ac and H3K9m3. These modifications were immunoprecipitated from pluripotent human embryonal carcinoma NCCIT cells by large scale Q^2^ChIP or by μChIP (1,000 cells). Notably, we set up a quantitative PCR (qPCR) approach and formulated an equation that enables assessment of chromatin fragmentation in small cell samples within a range of DNA fragment sizes suitable for ChIP. This was critical to ensure proper resolution of μChIP-chip analysis and, to our knowledge, is the only strategy to overcome this task with small cell numbers. Plotting average DNA fragment length against qPCR signal intensities from large scale sonicated samples reveals a linear relationship within the examined range (300-600 bp) of fragmentation (Additional data file 1). This is a useful average fragment size window for most ChIP applications. The linear relationship is described through the equation (y = 0.0012x - 0.0059), where y is the relative PCR signal intensity of the sample and × is the average DNA fragment length (Additional data file 1). Starting with 1,000 cells, sonication regimes of 3 × 30 s resulted in a relative PCR signal intensity of 0.502. The equation estimates an average DNA fragment length of approximately 420 bp; hence, this condition is suited for μChIP-chip analysis. Average fragment size was validated by agarose gel electrophoresis (Additional data file 1).

As part of the optimization of μChIP-chip, we carried out a comparison of different ChIP DNA isolation procedures. The phenol-chloroform isoamylalcohol DNA extraction method used in this study proved superior to the MinElute (QIAgen, Valencia, CA, USA; catalogue number 28004) and NucleoSpin Extract II (Machery-Nagel, Bethlehem, PA, USA; catalogue number 740609.10) DNA purification columns in that it recovered two to three times more DNA than the commercial kits, as determined by qPCR (data not shown). Hence, it enables a two- to three-fold further reduction in cell numbers for μChIP-chip relative to the commercial kits. Furthermore, an RNase digestion step was tailored to the fast downscaled ChIP procedure to remove RNA that would otherwise interfere with downstream amplification and array hybridization. Subsequently, DNA amounts in ChIP and input samples were measured with a Qubit fluorometer (Additional data file 2) to aid in determining the ChIP-DNA amplification conditions. DNA amount recovered from Q^2^ChIP inputs averaged 1,080 ng whereas μChIP inputs averaged 6.9 ng. These measurements were in line with an estimated DNA content of 6.6 pg per cell [15,16]. Q^2^ChIP recovered 3.2% and 4.3% of input DNA with antibodies to H3K9ac and H3K9m3, respectively (Additional data file 2). Higher recovery was observed with antibodies against H3K9m2 and H3K4m3 (data not shown), arguing that μChIP-chip is likely to also be effective with antibodies to other modified histones, which precipitate at least as well as those used here. DNA amounts in μChIP samples were estimated from Q^2^ChIP DNA recoveries and μChIP input to get a hint of the amount used for whole genome amplification (WGA). As determined by the assessed DNA amounts, Q^2^ChIP samples and inputs were amplified with the WGA2 kit (Sigma-Aldrich, St. Louis, MO, USA) whereas the WGA4 kit, optimized for very little DNA, was used for μChIP DNA amplification.

**Figure 2:**
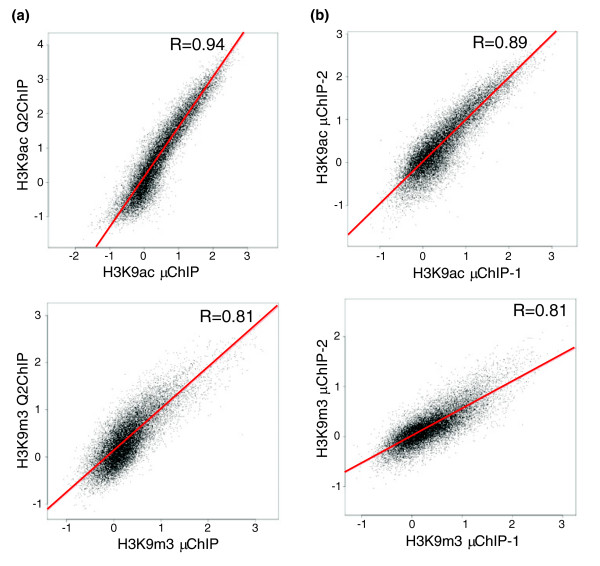
Reproducibility and specificity of μChIP-chip.** (a) **Two-dimensional scatter plots comparing μChIP-chip with Q^2^ChIP-chip using antibodies to H3K9ac and H3K9m3. **(b) **Comparison of two μChIP-chip experiments using antibodies to H3K9ac and H3K9m3. MaxTen values from each experiment are plotted on a log_2 _scale.

To assess the validity of small cell number ChIP-chip and the reproducibility of this assay, correlation analysis of log_2 _ChIP/input ratios between Q^2^ChIP-chip and μChIP-chip, and between replicates, was carried out with values resulting from Maxfour calculations [17]. This algorithm scores each promoter by finding the highest average log_2_ratio among ten consecutive probes per tiled region ('MaxTen'). To fully validate μChIP-chip, correlation analysis was carried out with MaxTen scores for all tiled regions, as opposed to the reported top 30% enriched promoters in an earlier study [13]. Both H3K9ac and H3K9m3 μChIP-chips robustly reproduced the large scale results (R = 0.80-0.94; Figure 2a). We then compared results from μChIP-chip biological replicates with each antibody to demonstrate high reproducibility (Figure 2b). Moreover, comparison between H3K9ac and H3K9m3 MaxTen values showed no correlation when assessed by large scale or μChIP-chip (data not shown), as expected from the mutually exclusive occupancy of these modifications.

### μChIP-chip and Q^2^ChIP-chip enrichment profiles for H3K9ac and H3K9m3

**Figure 3:**
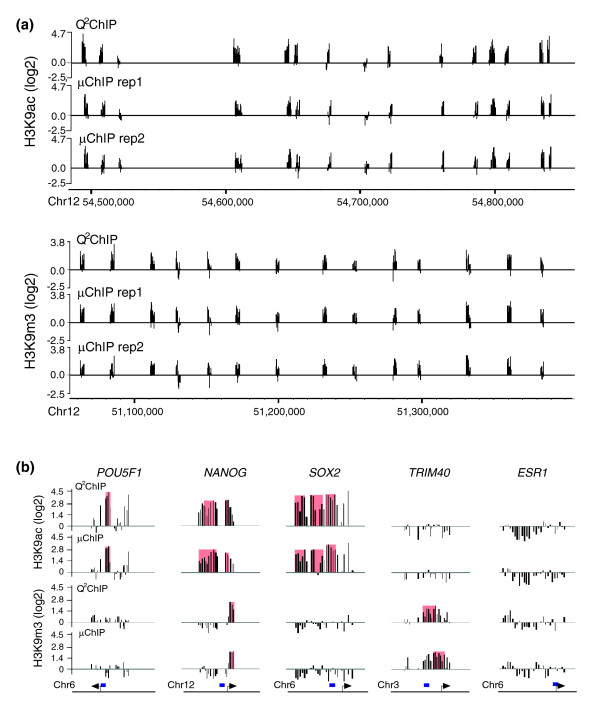
Enrichment profiles for H3K9ac and H3K9m3 expressed as log_2 _ChIP/input ratios in Q^2^ChIP-chip and μChIP-chip assays. **(a)** Comparison of enrichment profiles over a 360-kb region (H3K9ac) and a 300-kb region (H3K9m3) of chromosome 12 obtained by Q^2^ChIP-chip and in two μChIP-chip replicates. **(b) **Detailed profiles of H3K9ac and H3K9m3 enrichment on the 3-kb tiled regions (shown in bottom panels) of the *POU5F1*, *NANOG*, *SOX2*, *TRIM40 *and *ESR1 *promoters. Log_2 _ChIP/input ratios are shown in black and H3K9ac or H3K9m3 peaks (FDR ≤ 0.05) are superimposed (shaded in red). Tiled regions (bottom panel) also show the region examined by ChIP-qPCR (blue bars) in Figure 4.

Examples of normalized log_2 _ChIP/input signal ratios for two segments of chromosome 12 are shown in Figure 3a. For both H3K9ac and H3K9m3, the data show high similarity of enrichment profiles between μChIP-chip and Q^2^ChIP-chip, and high reproducibility of profiles between μChIP-chip replicates (Figure 3a). A detailed view of log_2 _ratios for tiled regions selected for their enrichment in either H3K9ac (*POU5F1 *and *SOX2 *promoters), H3K9m3 (*TRIM40*), both marks (promoter and exon 1 of *NANOG*), or none of these marks (*ESR1*), illustrates the high similarity of enrichment profiles also within a tiled region, and confirms the reproducibility between the two methods (Figure 3b). H3K9ac and H3K9m3 peaks detected with a false discovery rate (FDR) of ≤ 0.05 robustly overlapped between both ChIP assays (Figure 3b, red areas). Note, however, that due to limitations in the peak-calling software, the exact position of a peak may vary from array to array, and a broad peak may be called as a multiple peaks [17].

### Verification of μChIP-chip data by quantitative PCR

**Figure 4:**
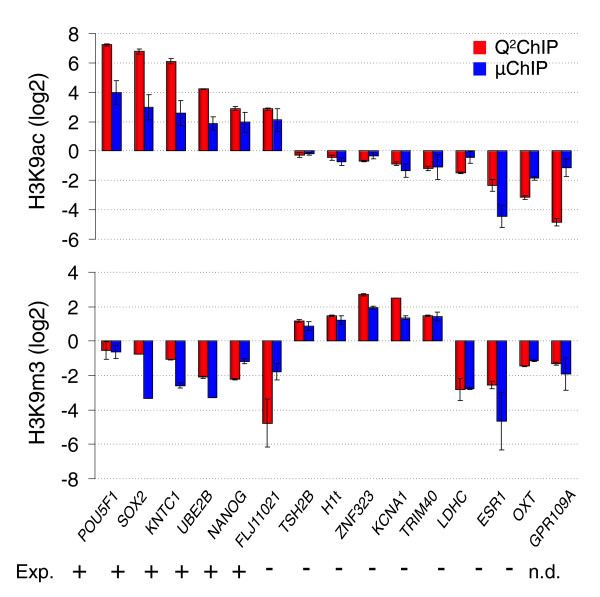
ChIP-qPCR analysis of H3K9ac and H3K9m3 association with the tiled regions of indicated genes. Experiments are based on the same cell batches as those examined by microarray. Data are expressed as log_2 _of fold-enrichment relative to input (mean ± standard deviation) in three independent experiments. Promoter regions covered by amplicons are shown in Figure 3b, bottom panels. The expression pattern of each gene was determined in duplicate transcriptome analysis of NCCIT cells using Affymetrix U133A GeneChips (not shown).

qPCR analysis of WGA-amplified μChIP DNA and non-amplified Q^2^ChIP DNA verified the array data (Figure 4). As expected from their expression in NCCIT cells, the *POU5F1*, *NANOG *and *SOX2 *promoters were acetylated on H3K9 in the absence of H3K9m3. The *UBE2B *housekeeping promoter was enriched in H3K9ac but not in H3K9m3, similarly to *KNTC1 *and *FLJ11021*, also expressed in NCCIT cells. Conversely, promoters of genes not expressed in NCCIT cells were either enriched in H3K9m3 without H3K9ac (*TSH2B*, *H1t*, *ZNF323*, *KCNA1*, *TRIM40*) or enriched in neither H3K9m3 nor H3K9ac (*LDHC*, *ESR1*, *OXT*, *GPR109A*) (Figure 4). Altogether, the data show that: promoters examined are enriched in either H3K9ac, H3K9m3 or none of these modifications, but not in both; active promoters are marked by H3K9ac in the absence of H3K9m3; and inactive promoters are marked by either H3K9m3 in the absence of H3K9ac, or by none of these marks, supporting the existence of both H3K9m3-dependent and independent gene repression mechanisms [18].

### Metagene analysis of H3K9ac and H3K9m3 enrichment

**Figure 5:**
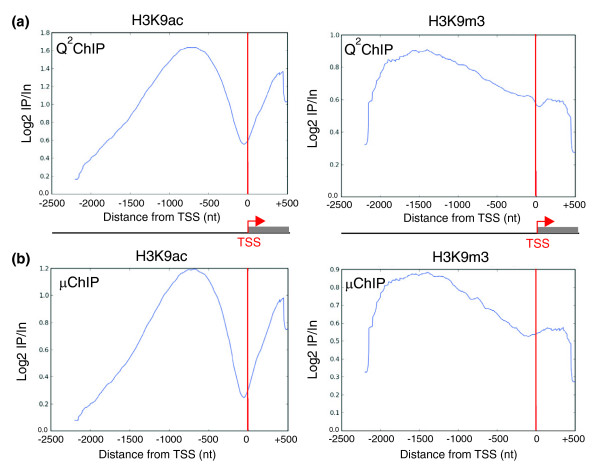
Metagene analysis of the distribution of H3K9ac and H3K9m3 in Q^2^ChIP-chip and μChIP-chip assays. H3K9ac and H3K9m3 occupancy profiles detected by **(a) **Q^2^ChIP-chip and **(b) **μChIP-chip in the 2.7 kb tiled regions. In (b), the same genes showing enrichment in either mark by Q^2^ChIP-chip (genes shown in (a)) were included in the analysis.

We next compared the average promoter enrichment profiles for H3K9ac and H3K9m3 over 2.7 kb within the tiled regions in large-scale ChIP-chip, and determined whether these profiles were maintained in μChIP-chip. We created a composite metagene from the collection of genes enriched (by the detection of one or more peaks with FDR ≤ 0.05; see Materials and methods) within the tiled region in either H3K9ac or H3K9m3 by Q^2^ChIP-chip. These two sets of genes were the basis for similar metagene analysis of μChIP-chip enrichment. Analysis of modified histone occupancy by large scale assays revealed distinct enrichment profiles for acetylated and trimethylated H3K9 (Figure 5a). H3K9ac showed a bimodal distribution with a pronounced dip immediately upstream of the TSS, suggesting a nucleosome-free region for active genes, as marked by acetylation; in contrast, H3K9m3 was more evenly distributed across the regions examined, with most prominent enrichment in the upstream half of the region and only a slight decrease in signal intensity around the TSS (Figure 5a). These profiles were rigorously conserved in μChIP-chip, both when we examined the same genes found to be enriched by either modification by Q^2^ChIP (Figure 5b), and when we examined all genes enriched in H3K9ac or H3K9m3 based on μChIP peak detection (data not shown). These findings support evidence that transcribed genes have a nucleosome-free region immediately upstream of the TSS, whereas most transcriptionally silenced genes do not [19]. H3K9m3-marked genes that lack a nucleosome-free region immediately 5' of the TSS may belong to a group of silent genes that do not recruit a pre-initiation complex, as the absence of pre-initiation complex recruitment in unexpressed genes has been shown to coincide with a lack of nucleosome depletion [20]. Members of a second and smaller group of silent genes do recruit a pre-initiation complex [20], and may be repressed by an H3K9m3-independent mechanism.

**Figure 6:**
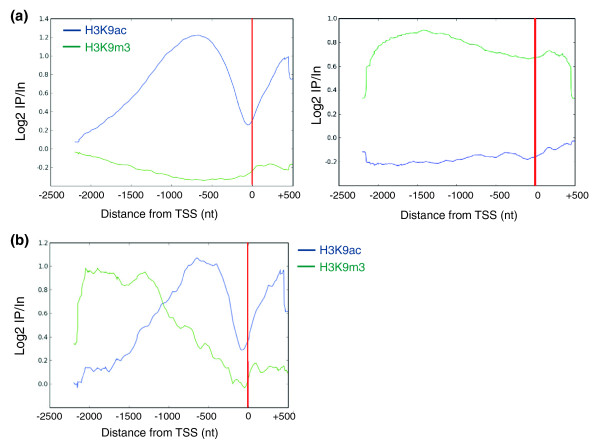
μChIP-chip identifies differential enrichment in H3K9ac and H3K9m3. **(a) **H3K9ac and H3K9m3 occupancy profiles detected by μChIP-chip on tiled regions with peaks in H3K9ac only (left) and H3K9m3 only (right). Genes containing at least one peak in H3K9m3 or H3K9ac, respectively, were removed from the analysis. **(b) **H3K9ac and H3K9m3 occupancy profiles exclusively on tiled regions containing both marks.

Distinct classes of genes based on differential enrichment in H3K9ac and H3K9m3 were evidenced by metagene profiles from μChIP-chip data on genes solely enriched in either H3K9ac (Figure 6a, left panel), H3K9m3 (Figure 6a, right panel) or both (Figure 6b) within the tiled region. Additionally, in concordance with the qPCR data, we identified a fourth group of genes not enriched in either of these modifications. We also found two groups of genes marked by H3K9ac: those devoid of H3K9m3 (Figure 6a, left panel) and those also enriched in H3K9m3 primarily upstream of the acetylated region (Figure 6b). Examination of individual genes confirmed the distribution of each mark throughout the tiled regions. H3K9m3 profiles also appear different based on the analyzed set of genes. Genes solely enriched in H3K9m3 display a relatively even distribution of this modification (Figure 6a, right panel), whereas genes also marked by H3K9ac show lower levels of H3K9m3 in the 3' half of the assessed region (Figure 6b). Metagene analysis of genes harboring both H3K9ac and H3K9m3 peaks within the tiled region reveals mutually exclusive occupancy profiles for these marks, and predominantly contain trimethylation upstream of the region marked by acetylation (Figure 6b). It will be interesting to investigate whether differential marking of genes by H3K9ac and H3K9m3 has implications on transcriptional regulation and hierarchy [21-23].

## Conclusion

Our results demonstrate that fast genome-scale analysis of promoter occupancy by modified histones is possible from as few as 1,000 cells. This represents an improvement over previous ChIP-chip protocols, which require significantly more cells and take at least 4 days up to hybridization [13,24]. Specific steps of the Acevedo *et al*. [13] microChIP protocol that differ from our μChIP-chip assay are detailed in Additional data file 3. Although performant, in its current form μChIP-chip has limitations. First, we cannot formally exclude that the DNA amplification step does not introduce bias. A recent comparison of three widely used amplification procedures, including WGA, reports that all procedures introduce some bias [25]. Notably however, WGA-based amplification resulted in the most accurate performance. We show here by qPCR that amplification of ChIP DNA from 1,000 cells introduces little bias; however, when establishing μChIP-chip, amplification of DNA from 100 cells produced inconsistencies, presumably due to lower signal/noise ratios [12] and more rounds of amplification were required owing to the minute amount of template DNA used. Development of improved amplification protocols may result in successful application of μChIP-chip to fewer than 1,000 cells. Secondly, μChIP-chip may also be suitable for non-histone proteins, although this remains to be tested. For analysis of low-abundance or transiently bound proteins, cell numbers might need to be increased compared to histone μChIP-chip. Further, we formulate an equation that allows an estimation of the average DNA fragment length produced by sonication of chromatin from minute cell samples. It is imperative to assess chromatin fragmentation prior to ChIP location analysis to ensure good resolution and valid analysis. To our knowledge, this is the only strategy to overcome this task with small cell numbers. Moreover, array data show high reproducibility between biological replicates and conservation of H3K9ac and H3K9m3 enrichment profiles in the large scale and μChIP-chip assays. We demonstrate that μChIP-chip can be applied to reveal nucleosome-free regions in as few as 1,000 cells. In addition, metagene analysis reveals distinct occupancy profiles for each histone modification in the tiled regions, which are maintained in μChIP-chip, and identify four distinct groups of genes in human embryonal carcinoma cells. μChIP-chip therefore makes genome-wide epigenetic analyses amenable to small cell samples, such as rare stem cell subpopulations, cells from the early embryo or human biopsies.

## Materials and methods

### Materials

Pluripotent human embryonal carcinoma NCCIT cells were cultured as described [10]. Antibodies against H3K9ac were from Upstate (Millipore Inc., Billerica, MA, USA; catalogue number 06-942) and antibodies to H3K9m3 were from Diagenode (Liège, Belgium; catalogue number pAb-056-050). All other reagents were from Sigma-Aldrich unless otherwise indicated.

### ChIP assays

The Q^2^ChIP assay, referred to as large scale ChIP, was performed as described [10]. Chromatin was prepared from 2 × 10^6 ^cells, diluted to 2 A_260 _units and aliquoted into 100 μl per ChIP.

μChIP was carried out from 1,000 cells per ChIP [11,12] with modifications and optimization to enable genome-wide analysis. A troubleshooting guide is presented in Additional data file 4. Primary antibodies (2.4 μg) were coupled to Dynabeads Protein A (10 μl; Dynal Biotech, Invitrogen, Oslo, Norway) in RIPA buffer (10 mM Tris-HCl, pH 7.5, 1 mM EDTA, 0.5 mM EGTA, 1% Triton X-100, 0.1% SDS, 0.1% Na-deoxycholate, 140 mM NaCl) for 2 h at 4°C. Tubes were in strips handled in a magnetic rack (Diagenode, catalogue number kch-816-001). Concomitantly, cells were detached by a few sharp blows to the flask in the presence of 20 mM of the histone deacetylase inhibitor sodium butyrate, and aliquots of 1,000 cells were suspended in 500 μl phosphate-buffered saline/butyrate. Proteins and DNA were cross-linked with 1% formaldehyde for 8 minutes and cross-linking was stopped with 125 mM glycine. Cells were centrifuged at 620 g in a swing-out rotor for 10 minutes at 4°C and washed twice in 0.5 ml ice-cold phosphate-buffered saline/butyrate by gentle vortexing and centrifugation as above. Approximately 20 μl buffer was left with the pellet after removal of the last wash. Cells were lysed by addition of 120 μl room temperature lysis buffer (50 mM Tris-HCl, pH 8, 10 mM EDTA, 1% SDS, protease inhibitor cocktail (Sigma-Aldrich, catalogue number P8340), 1 mM PMSF, 20 mM butyrate) and thorough vortexing. Following a 3-minute incubation on ice and additional vortexing, nuclei were centrifuged at 860 g for 10 minutes and the supernatant discarded, leaving approximately 20 μl lysis buffer in the tube. RIPA buffer (120 μl containing protease inhibitor cocktail, 1 mM PMSF, 20 mM butyrate) was added, the tube was vortexed thoroughly and cells sonicated for 3 × 30 s on ice with 30 s pauses, using a probe sonicator (Labsonic-M, 3-mm probe; cycle 0.5, 30% power; Sartorius AG, Göttingen, Germany) to produce fragments of approximately 400-500 bp. The sample was centrifuged at 12,000 g for 10 minutes at 4°C and the supernatant was transferred into a 0.2-ml PCR tube containing beads pre-incubated with antibodies, leaving approximately 10 μl of supernatant behind. A chromatin sample identical to that used in the ChIP sample was prepared to represent the input and transferred into a 1.5 ml tube.

Proper chromatin fragmentation from 1,000 cells was ensured using a qPCR assay further developed from that previously described [12]. Since the reliability of ChIP depends on control of chromatin fragmentation, we accomplished this for our genome-scale location analysis by formulating an equation that enables an estimation of average fragment length in minute cell samples. A linear relationship between average DNA fragment length and qPCR signal intensities within a fragmentation range useful for ChIP (300-600 bp) was described through this equation, which allows calculation of DNA fragment length after experimental determination of the relative PCR signal in a sonicated 1,000-cell sample (Additional data file 1).

Immunoprecipitation and washes of the ChIP product were performed essentially as described [12]. Beads were released into the chromatin suspension and rotated at 40 rpm for 2 h at 4°C. The ChIP material was washed three times by 4-minute incubations in 100 μl of RIPA buffer and once in 100 μl of 10 mM Tris-HCl, pH 8.0, 10 mM EDTA (TE) buffer, and transferred into a new tube while in TE buffer. Elution buffer (20 mM Tris-HCl, pH 7.5, 5 mM EDTA, 20 mM butyrate, 50 mM NaCl, 1% SDS) (150 μl) and 5 μg RNase (Roche, Basel, Switzerland; catalogue number 11119915001) were added after removal of TE buffer. The same amount of RNase was added to the input, and ChIP and input samples were incubated at 37°C for 20 minutes on a Thermomixer (1,300 rpm; Eppendorf, Hamburg, Germany). Samples were briefly centrifuged, 1 μl of proteinase K (at 20 μg/μl) was added and tube lids were replaced by new ones to prevent leakage resulting from softening of the plastic upon heating. DNA elution, cross-link reversal and proteinase K digestion were carried out in a single step for 2 h at 68°C on a Thermomixer. After capturing of beads, the supernatant was recovered, beads were incubated for another 5 minutes in 150 μl elution buffer containing 50 μg/ml proteinase K, and both supernatants were pooled. ChIP and input samples were made up to a final volume of 490 μl in elution buffer without SDS. ChIP DNA was extracted with phenol-chloroform isoamylalcohol and chloroform isoamylalcohol, ethanol-precipitated in the presence of 10 μl acrylamide carrier (Sigma-Aldrich, catalogue number A9099) and dissolved in 10 μl MilliQ water.

### Whole genome amplification and clean up of ChIP DNA

ChIP and input DNA were amplified with WGA2 (Q^2^ChIP) or WGA4 (μChIP) GenomePlex Whole Genome Amplification Kits (Sigma-Aldrich) as per the manufacturer's instructions; however, we omitted the lysis and DNA fragmentation steps. Starting from step 6 in the WGA procedures, library preparation was carried out and immediately followed by amplification for 14 or 25 cycles for Q^2^ChIP and μChIP, respectively.

Amplified DNA was cleaned up using the QIAquick PCR purification kit (Qiagen, catalogue number 28104) as per the manufacturer's instructions except that five volumes of buffer PB (Qiagen, catalogue number 19066) were used instead of buffer PBI to ensure the absence of pH indicator in the sample (the pH indicator in buffer PBI may interfere with microarray applications). Furthermore, DNA was eluted in 30 μl 1 mM Tris-HCl, pH 8.0. The kit is designed for purification of DNA fragments of 100-10,000 bp, and thus was well suited for ChIP and input DNA fragments. Following DNA purification, samples were quantified by NanoDrop (NanoDrop Technologies, Wilmington, DE, USA) and aliquots were diluted to 7.5 ng/μl in TE buffer for PCR-based quality assessment. Importantly, parallel ChIP experiments were carried out without amplification and were directly assessed by qPCR to serve as a reference for amplified samples as well as for array data. Quality of amplified samples was also evaluated by agarose gel electrophoresis. Typically, amplification produced 7.5-15 μg DNA (depending on the WGA kit lot number) with an average size of approximately 400-500 bp. WGA amplification can therefore yield enough DNA to probe as many as seven arrays without further amplification.

### DNA labeling and array hybridization

ChIP and input DNA fragments were labelled with Cy5 and Cy3, respectively, and hybridized onto Nimblegen human HG18 RefSeq Promoter arrays. Arrays covered approximately 27,000 human RefSeq promoters, ranging from -2,200 to +500 bp relative to the transcription start site (TSS). Probes consisted of 385,000 50- to 75-mers tiled throughout non-repetitive genomic sequences at a median spacing of 100 bp. Sequence source for probes was the UCSC Genome Browser. ChIP and input DNA labeling, hybridization and detection were performed using the services of Nimblegen (Madison, WI, USA).

### Data analysis

Signal intensity data were extracted from the scanned images of each array using NimbleScan software. Log_2 _ChIP/input ratios were scaled and centered around zero by subtracting the bi-weight mean for the log_2 _ratio values for all features on the array from each log_2 _ratio value. Peaks were detected by searching for four or more probes with a signal above a cut-off value using a 500-bp sliding window. Cut-off values were a percentage of a hypothetical maximum defined as (mean + 6 [standard deviation]). Ratio data were randomized 20 times to evaluate the probability of false positives, and each peak was assigned a FDR score. Normalization and peak detection were performed by Nimblegen in accordance with their protocols. This process uses a cut-off range of 90% to 15%, with higher cut-offs corresponding to more stringent peak detection, as reflected in the FDR calculation. The Nimblegen protocol was recently evaluated as part of a comprehensive study that objectively analyzed the performance of a number of commercially available ChIP-chip array platforms and signal detection algorithms [26], and found to deliver reliable results.

For scoring the promoters before correlation analysis, we assigned an amplification value to each promoter by applying the Maxfour algorithm with a ten-probe window [27]. For each promoter, the corresponding probes' log_2 _ratios were scanned in genome order with a ten-probe window. The highest ten-probe average was used as the amplification value for the promoter. Promoters represented by less then ten probes (1.5% of the total) were not included in the analysis.

### Metagene analysis of regions containing H3K9ac or H3K9m3

Metagene analysis of promoter occupancy was performed essentially as described [28]. Genes with a high probability of enrichment (FDR ≤ 0.05) in H3K9ac or H3K9m3 marks within the tiled region were collected and used to assemble a metagene of the average composite binding. Each region was interrogated for probes and these were mapped into a 2.7-kb wide window at the appropriate offsets based on strand orientation. Linear interpolation was used to estimate the fold enrichment at each base position within the 2.7-kb window. This interpolation left the 5' and 3' ends of the window under-represented. The metagene was created from this collection of functions by calculating the mean of the values mapped to each position by all the regions found to be enriched in H3K9ac or H3K9m3 Q^2^ChIP or μChIP. If the offset corresponded to the exact location of a probe within a specific tiled region, values were directly measured; if not, values were linearly interpolated from the values of the two flanking probes [28]. Genes solely enriched by only one of the examined marks were selected from the entire set of genes harboring the mark (peak detection with FDR ≤ 0.05) and then removing from that set all genes also possessing a peak for the other mark.

### Quantification of non-amplified ChIP DNA

Because the NanoDrop spectrophotometer does not allow accurate quantification of minute amounts of non-amplified ChIP DNA, we used a Qubit fluorometer (Invitrogen, Carlsbad, CA, USA; catalogue number Q32857) and a Quant-iT dsDNA HS kit (Invitrogen, catalogue number Q32851) for quantification. Ten percent of Q^2^ChIP DNA samples and whole μChIP inputs were mixed with Quant-iT working solution to a final volume of 200 μl, incubated for 2 minutes and analyzed by the Quant-iT DNA HS program on a Qubit fluorometer.

### Quantitative PCR

Immunoprecipitated DNA from three independent ChIPs was analyzed by duplicate qPCR [10] (Additional data file 5). qPCR data are expressed as mean (± standard deviation) log_2 _values of enrichment relative to input DNA.

## Abbreviations

ChIP: chromatin immunoprecipitation; FDR: false discovery rate; H3K9ac: acetylated lysine 9 of histone H3; H3K9m3: trimethylated lysine 9 of histone H3; μChIP: microChIP; qPCR: quantitative PCR; TSS: transcription start site; WGA: whole-genome amplification.

## Authors' contributions

JAD designed the study, performed experiments, contributed to analysis design, made figures and wrote parts of the manuscript. AHR performed bioinformatics analyses, made figures and wrote parts of the methods. PC designed the study, wrote the manuscript, made figures and supervised the work. All authors read and approved the final manuscript.

## Additional data files

The following additional data are available with the online version of this paper. Additional data file 1 is a figure describing the steps behind the equation formulated to estimate, using qPCR, chromatin fragment length after a given sonication regime of 1,000 cells. Additional data file 2 a table providing values of DNA recovery from Q^2^ChIP and μChIP. Additional data file 3 is information on a technical comparison between μChIP-chip and a previously published protocol. Additional data file 4 is a troubleshooting guide for μChIP-chip. Additional data file 5 is a table listing ChIP qPCR primers used in this study.

## Supplementary Material

Additional data file 1 Steps behind the equation formulated to estimate, using qPCR, chromatin fragment length after a given sonication regime of 1,000 cells. Click here for file

Additional data file 2 DNA recovery from Q^2^ChIP and μChIP. Click here for file

Additional data file 3 Technical comparison between μChIP-chip and a previously published protocol. Click here for file

Additional data file 4 Troubleshooting guide for μChIP-chip. Click here for file

Additional data file 5 ChIP qPCR primers used in this study. Click here for file
