# Supplementary material for: Fast genomic μChIP-chip from 1,000 cells
Source: Genome Biol. 2009 Feb 10;10(2):R13. doi: 10.1186/gb-2009-10-2-r13 (PMC2688267; doi:10.1186/gb-2009-10-2-r13)
Supplement: Additional data file 1 — Steps behind the equation formulated to estimate, using qPCR, chromatin fragment length after a given sonication regime of 1,000 cells. [file gb-2009-10-2-r13-S1.doc]

**Fast genomic ChIP-chip from 1,000 cells**

John Arne Dahl, Andrew Reiner and Philippe Collas

**Additional data file #1**


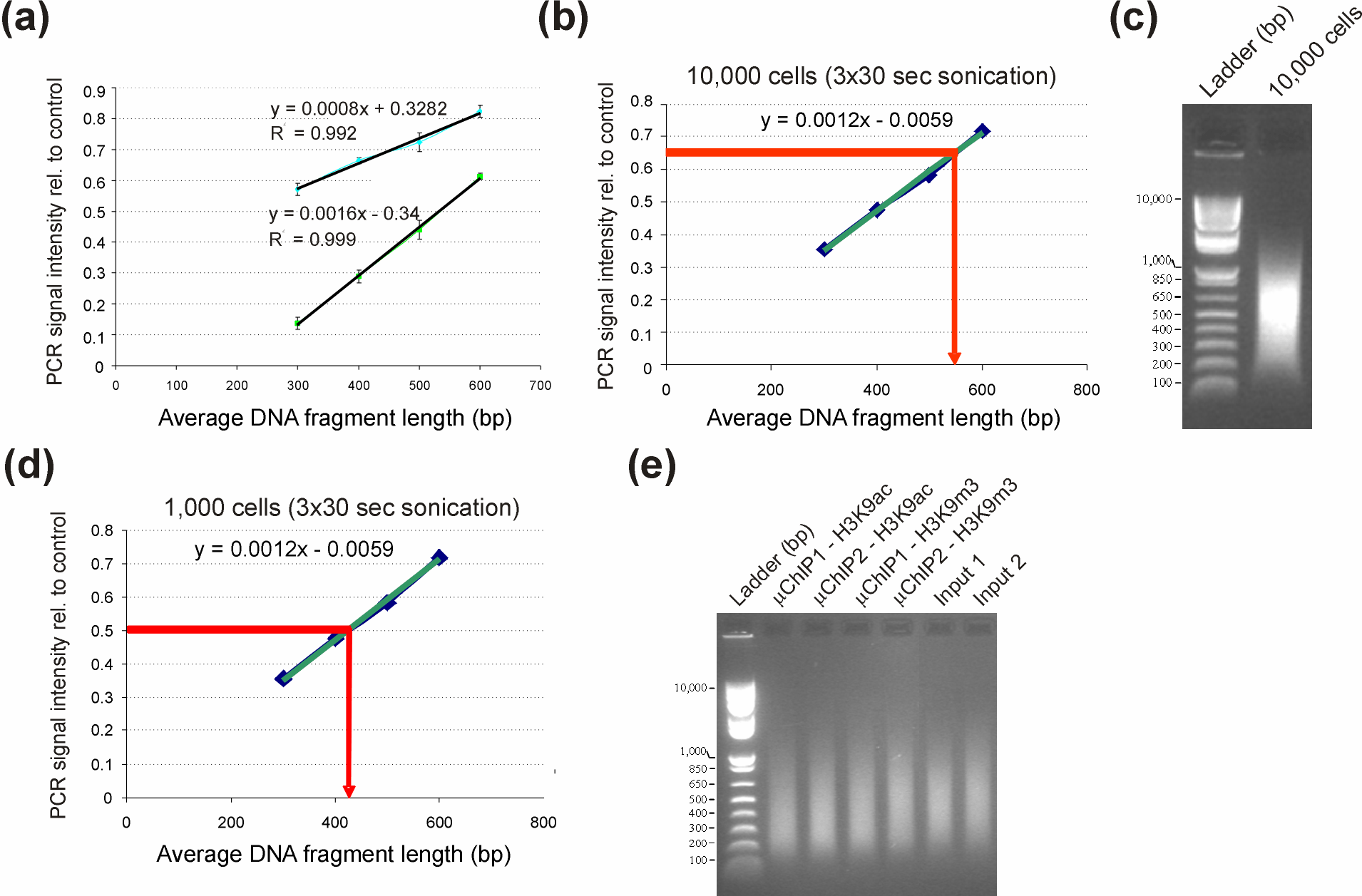


Assessment of chromatin fragmentation by real time qPCR. Quantitative PCR was carried out on DNA from 100,000-cell batches of sonicated chromatin with an average DNA fragment length of either 300, 400, 500 or 600 bp, as determined by agarose gel electrophoreses. Signal strength from intact (non-sonicated) DNA from 100,000 cells was used as reference (1.0 value on the y axis). Relative PCR signal intensity for the batches of fragmented DNA was determined by the ratio of [PCR signal sonicated sample] / [PCR signal non-fragmented control]. Amplicons of 298 and 96 bp were used (Dahl and Collas, 2008. Nat. Protoc. 3, 1032-1045).

**(a)** Plotting of relative PCR signal intensity against average DNA fragment length reveals a linear relationship for both amplicons within the presented range of fragmentation (96 bp signal intensities in blue, 298 bp signal intensities in green and linear regression lines in black. Equations describing the linear relationship are shown). The linear range and the exact equation describing the linear relationship is likely to vary with the size of the amplicon and must be experimentally assessed for each amplicon. The linearity can not be extrapolated to describe the average DNA fragment length for outside the assessed range, as such claims would argue that an average fragment length of 900 bp would result in a relative PCR signal of 1. Such fragmentation would undoubtedly disrupt some templates, reducing the relative PCR signal. Furthermore, a relative PCR signal for the 96 bp amplicon of 0.328 (as is where the line of the 96 bp based equation would cross the y-axes) intuitively can not come from a sample with an average DNA fragment length of ~0 bp. Moreover, for the 298 bp based equation, an average DNA fragment length of ~210 bp (as is where the line of this equation would cross the x-axes) would not result in a PCR signal of 0 as in reality the sonicated DNA fragments vary in size rather than have a defined length. Hence, some templates are likely to be intact. However, and despite this limitation, the identified average fragment size window for which we show a linear relationship between average DNA fragment length and relative PCR signal intensities is a useful average fragment size window for most ChIP applications.

(**b**) Signal intensities from the two amplicons were averaged to give a linear relationship described by a single equation, allowing for direct estimation of the average of the two DNA fragment length-estimates obtained by two separate equations (shown in **a**). The linear relationship described in (**a**) can be used to estimate average DNA fragment length for a small number of cells after sonication treatment. Following experimental determination of the PCR signal of purified DNA from a sonicated cell sample and the PCR signal from intact (non-sonicated) DNA obtained from an equal number of cells, the relative PCR signal intensity of the sonicated sample is calculated. This relative PCR signal intensity can be plotted into the equation describing the linear relationship to estimate average DNA fragment length of the sonicated sample. However, and importantly, if the relative PCR signal falls outside the range of the deduced linear relationship, sonication conditions must be adjusted until the relative PCR signal is within this range. As shown for a 3 x 30 sec sonication regime for 10,000 cells, the combined equation (y = 0.0012x – 0.0059) estimates an average fragment length of ~540 bp (regression line in green, in **b**). Separate estimation by each of the two equations in (**a**) gives ~530 and ~550 bp, an average of ~540 bp, for the 96-bp and 298-bp amplicons, respectively.

(**c**) Validation of equation based estimation by agarose gel electrophoreses of DNA from a pool of 15 batches of 10,000 cells, each sonicated for 3 x 30 sec.

(**d**) The equation of the regression line was applied to determine a suitable fragmentation condition for 1,000 cells based on the PCR signal intensities of non-sonicated and sonicated 1,000-cell samples. The red arrow illustrates the read-out from 3 x 30 sec sonication regimes where the average relative PCR signal intensity from the 298- and 96-bp amplicons was 0.502±0.035. The equation estimates an average DNA fragment length of ~420±28 bp, hence this condition is suited for ChIP analysis. Separate estimation through each of the two equations shown in (**a**) gives for the 96 bp-amplicons ~390±25 bp, and for the 298 bp-amplicons ~450±31 bp. Data are the average±SD of two independent experiments, each assessed by duplicate qPCR for both amplicons.

(**e**) Validation of equation based estimation of average DNA fragment length by agarose gel electrophoreses of WGA4 amplified DNA from duplicate µChIP samples and duplicate inputs. Visualization of average DNA fragment lengths resulting from sonication regimes of 3 x 30 sec applied to 1,000 cells is in line with the estimation in (**d**), in that the average fragment length is ~400-500 bp. Further validation of the fragmentation by the sonication regime applied for 1,000 cells comes from comparison to fragmentation of 10,000 cells with the same sonication regime. This is based on the rationale that chromatin from 1,000 cells is as, or more, fragmented by equal sonication energy as a sample containing ten times more chromatin. To support this view, Since 10,000 cells are fragmented to ~540 bp, it is very much as expected that fragmentation of 1,000 cells is estimated to ~420±28 bp.
