# Supplementary material for: Fast genomic μChIP-chip from 1,000 cells
Source: Genome Biol. 2009 Feb 10;10(2):R13. doi: 10.1186/gb-2009-10-2-r13 (PMC2688267; doi:10.1186/gb-2009-10-2-r13)
Supplement: Additional data file 2 — DNA recovery from Q2ChIP and μChIP. [file gb-2009-10-2-r13-S2.doc]

**Additional data file #2**

DNA recovery from Q^2^ChIP and µChIP^a^.

| Sample | Q^2^ChIP  (ng DNA) | µChIP  (ng DNA) |
| --- | --- | --- |
| Input | 1080±79 | 6.9±0.4 |
| H3K9ac | 34 ± 1.3 (3.2 %) | 0.23 (3.2%) |
| H3K9m3 | 46 ± 1.8 (4.3 %) | 0.31 (4.3%) |
| No-antibody control | 14 ± 0.5 (1.3 %) | 0.09 (1.3%) |

^a^ DNA amounts in Q^2^ChIP input and ChIP samples and µChIP input samples were measured using a Qubit fluorometer. Based on these measurements, DNA amounts in µChIP samples were estimated. Note that these measurements are probably an underestimate, considering the previously reported locus-specific increase in relative precipitation upon reduction in cell numbers. Data are the average ± SD of 3-4 independent experiments. Percent of input value is also shown.
