# Supplementary material for: Fast genomic μChIP-chip from 1,000 cells
Source: Genome Biol. 2009 Feb 10;10(2):R13. doi: 10.1186/gb-2009-10-2-r13 (PMC2688267; doi:10.1186/gb-2009-10-2-r13)
Supplement: Additional data file 3 — Technical comparison between μChIP-chip and a previously published protocol. [file gb-2009-10-2-r13-S3.doc]

**Additional data file #3**

Key differences between the microChIP-chip assay of Acevedo et al. (Biotechniques 43, 791-797) and the μChIP-chip assay reported here.

Improved detection limit and rapidity of our µChIP-chip assay compared to the miniaturized protocol of Acevedo et al. is likely due to a number of differences.

*• Use of Dynabeads protein A versus Staph A cells*. Acevedo et al. use Staph A cells for binding immune complexes; µChIP-chip uses Dynabeads Protein A, eliminating time of preparation of Staph A cells. Additionally, Staph A cells are treated overnight with herring sperm DNA and bovine serum albumin to reduce unspecific binding. In contrast, Dynabeads Protein A shows very little propensity to unspecific binding, making precoating unnecessary. Testing herring sperm DNA and BSA protein blocking of Dynabeads Protein A showed no difference in unspecific binding relative to untreated beads in our hands. However, such blocking reduced specific binding by ~20% (data not shown). Avoiding blocking with herring sperm DNA eliminates the risk of contamination that might be amplified alongside sample DNA and affect downstream analysis. Further, the paramagnetic nature of Dynabeads Protein A allows rapid handling of the samples in a magnetic stand instead of time consuming centrifugations. Lastly, magnetic capture of the beads on the tube wall leads to more efficient washing and reduced sample loss than having a pellet at the bottom of the tube.

*• Precoating of antibodies to beads*. Acevedo et al. first incubate chromatin with antibodies, and subsequently with Staph A cells. Thus, excess (unbound) antibodies compete with chromatin-antibody complexes for binding to Staph A cells. Precoating of Dynabeads Protein A with antibodies prior to incubation with chromatin, as outlined in our procedure, ensures maximum binding of chromatin target.

*• Risk of sample loss due to exposure to surfaces*. Acevedo et al. report extensive sample exposure to (plastic) surfaces through transfers to new tubes and pipetting. This occurs, for example (i) in the preclearing of chromatin by Staph A cells, (ii) in the transfer of the chromatin supernatant to a clean tube after preclearing chromatin with Staph A cells, (iii) in the resuspension of immune complexes with a pipette tip for each of the 6 washes and (iv), after elution of precipitated chromatin, upon two subsequent transfers of the supernatant to new tubes, to remove Staph A cells. Contrary to this, µChIP-chip limits sample loss through surface exposure to a minimum.

*• DNA purification method*. Acevedo et al. purify ChIP DNA using QIAquick MINelute columns. We report in the present paper that this recovers 2-3-fold less DNA than phenol-chloroform isoamylalcohol extraction.

*• Buffers used*. Another variation between the two ChIP assays is the composition of various buffers. On a speculative note, relatively increased detection limit of µChIP-chip may originate from the higher stringency of its ChIP incubation and wash buffers. However, the complexity of the buffers used in both assays necessitates experimental comparison to address this issue.

*• Shortening and combination of steps.* Other steps behind increased rapidity of µChIP-chip are, for instance, reducing the overnight incubation step of antibody binding to chromatin to 2 h, completing washing by 4 rounds as opposed to 6, and carrying out cell lysis in a single step instead of separate lysis of cells and nuclei. In summary, the µChIP-chip assay includes shortening and combination of steps that altogether accelerate the procedure.
