# Supplementary material for: Fast genomic μChIP-chip from 1,000 cells
Source: Genome Biol. 2009 Feb 10;10(2):R13. doi: 10.1186/gb-2009-10-2-r13 (PMC2688267; doi:10.1186/gb-2009-10-2-r13)
Supplement: Additional data file 4 — Troubleshooting guide for μChIP-chip. [file gb-2009-10-2-r13-S4.doc]

**Additional data file #4**

Troubleshooting guide

| **Problem** | | **Possible cause** | **Remedy** |
| --- | --- | --- | --- |
| No PCR signal from ChIP samples | Amount of chromatin in the assay is insufficient | | Increase number of cells or amount of chromatin. It may be difficult to extract all chromatin from specific cell types (e.g., chondrocytes). |
|  | ChIP does not work | | Use ChIP-grade antibodies. Carry out a titration of the antibodies. |
|  | PCR does not work | | Carry out a control qPCR with the same primers on genomic DNA and optimize PCR conditions. |
| ChIP PCR gives signal weaker than anticipated | Amount of DNA template in PCR reaction is insufficient | | Increase the amount of ChIP DNA template in the PCR. Increase the amount of chromatin in the ChIP assay. |
| Insufficient chromatin fragmentation | Material is over cross-linked | | Reduce duration of cross-linking. |
|  | Cell lysis is insufficient. | | Increase vortexing time and intensity. Snap-freeze in liquid nitrogen and thaw to facilitate lysis. Assess lysis under the microscope. |
|  | Insufficient sonication. | | Increase sonication time and/or number of sonication rounds. If using a probe sonicator, make sure the probe is not in contact with the tube wall. |
|  | Foaming occurs during sonication. | | Ensure the sonicator probe is placed deep enough in a sample. Reduce sonication intensity. |
| High background signal | Immunoprecipitation is too unspecific. | | Use ChIP-grade antibodies. Titrate antibody. Reduce antibody/bead incubation time with chromatin. Make sure tube-switch step is carried out. |
|  | Washes are insufficient. | | Increase number and/or stringency of washes after the immunoprecipitation. |
| PCR signal intensity varies between replicates | Chromatin preparations are inconsistent between samples. | | Remove large insoluble chromatin clumps and cell debris by centrifugation after fragmentation. Make sure not to carry-over debris when aspirating the chromatin supernatant. |
|  | Sonication outcomes are inconsistent. | | Practice sonication on larger cell numbers (e.g. 100,000) until fragmentation is reproducible. |
|  | Amount of Dynabeads is inconsistent between samples. | | Ensure the magnetic beads are well suspended while pipetting bead/antibody conjugates into the chromatin samples. |
|  | Amounts input DNA templates are too low and too variable, producing high Ct values. | | Increase amount of input DNA template and ensure consistency between replicates. Make sure the precipitated ChIP DNA is fully dissolved before using it for PCR. |
| Less amplified DNA obtained than anticipated | DNA is degraded. | | Go directly from library preparation to the amplification step. |
|  | WGA kit is defective. | | Test kits with sonicated “input” (not ChIP) DNA before using it on ChIP samples. Use new kit with new batch number that produces sufficient amounts of DNA. Note that we have experienced variations in WGA kits, resulting in variable outputs. |

**Note:** For those new to ChIP we recommend practicing the assay with larger cell numbers (e.g. 100,000 cells) and qPCR until high reproducibility is achieved, before setting up experiments with 1,000 cells for array analyses.
