# Supplementary material for: Fast genomic μChIP-chip from 1,000 cells
Source: Genome Biol. 2009 Feb 10;10(2):R13. doi: 10.1186/gb-2009-10-2-r13 (PMC2688267; doi:10.1186/gb-2009-10-2-r13)
Supplement: Additional data file 5 — ChIP qPCR primers used in this study. [file gb-2009-10-2-r13-S5.doc]

**Additional data file #5**

Quantitative ChIP PCR primers used in this study and genomic localization of the amplicons.

| Gene | Chr.  location | Forward primer (F)  Reverse primer (R) | Annealing  temp. (^o^C) | Product  size (bp) |
| --- | --- | --- | --- | --- |
| *ESR1* | 6q24-q27 | F: TGTGCTCTTTTTCCAGGTGGC^a^  R: TCGTTCCCTTGGATCTGATGC^a^ | 60 | 151 |
| *FLJ11021* | 12q24.31 | F: ACTCTGGCGGCGGAATGC  R: CAGCGGAGAGCGTTTAAGTATGG | 60 | 99 |
| *GPR109A* | 12q24.31 | F: CTCCTTGCTGGAGCATTCAC^a^  R: GGCAACACCTTGACAATGAA^a^ | 60 | 149 |
| *H1t* | 6q22.1 | F: CAGCACTAGACCCTGGGAGA^a^  R: TACACCAGCACTGGCAGAAG^a^ | 60 | 255 |
| *KCNA1* | 12p13 | F: CACCTGGGATCAGGAAGAAA^a^  R: TGCCTTTCACTCAGTTTGGA^a^ | 60 | 133 |
| *KNTC1* | 12q24.31 | F: GGAACTACCAATTCTGACTGATGC  R: ACTGTAACGCTTCTCGGACTTC | 60 | 81 |
| *LDHC* | 11p15.1 | F: GCGTTTCATTACCACCCTCT^a^  R: GTCCAGATCACCGAAATGCT^a^ | 60 | 182 |
| *NANOG* | 12q13.31 | F: GTTCTGTTGCTCGGTTTTCT  R: TCCCGTCTACCAGTCTCACC | 60 | 95 |
| *OXT* | 20p13 | F: AAGGCACCTCACCTTCTGTG^a^  R: TCGGTGGAGCTCTGTTTAAGA^a^ | 60 | 163 |
| *POU5F1* | 6q21.33 | F: AGTCTGGGCAACAAAGTGAGA  R: AGAAACTGAGGAGAAGGATG | 60 | 169 |
| *SOX2* | 3q26.3-q27 | F: GAGAAGGGCGTGAGAGAGTG ^b^  R: AAACAGCCAGTGCAGGAGTT ^b^ | 60 | 153 |
| *TRIM40* | 6p21.31 | F: AGTGACAGCCCTTATTAGTG  R: AAGAATGGACATCCTAGAACC | 60 | 188 |
| *TSH2B* | 6q22.2 | F: CAGACATCTCCTCGCATCAA^a^  R: GGAGGATGAAAGATGCGGTA^a^ | 60 | 250 |
| *UBE2B* | 5q31.1 | F: CTCAGGGGTGGATTGTTGAC^a^  R: TGTGGATTCAAAGACCACGA^a^ | 60 | 177 |
| *ZNF323* | 6p | F: GGTGGTTCTGTTCTTGAC  R: GAAGGAGTAGATTAGGAAGC | 60 | 219 |

^a^ From Weber et al., 2007. Nat. Genet. 39, 457-466.

^b^ From Takahashi et al., 2007. Cell 131, 861-872.
